# Outcomes of Open, Laparoscopic, and Percutaneous Drainage of Infected Walled-Off Pancreatic Necrosis: A Nationwide Inpatient Sample Study

**DOI:** 10.7759/cureus.12972

**Published:** 2021-01-28

**Authors:** Jeffrey Rebhun, Najib Nassani, Alex Pan, Mindy Hong, Asim Shuja

**Affiliations:** 1 Department of Internal Medicine, University of Illinois at Chicago, Chicago, USA; 2 Department of Gastroenterology and Hepatology, University of Illinois at Chicago, Chicago, USA; 3 Department of Dermatology, Northwestern University Feinberg School of Medicine, Chicago, USA

**Keywords:** pancreas, infected pancreatic necrosis, walled-off pancreatic necrosis, acute pancreatitis complications, pancreatic intervention

## Abstract

Background

Walled-off pancreatic necrosis (WOPN) represents an encapsulated collection of necrotic pancreatic or peripancreatic tissue that tends to develop four weeks after the onset of acute necrotizing pancreatitis. When infected, it is managed initially by antibiotic therapy before drainage by endoscopic, percutaneous, or surgical means. This study aims to describe the morbidity, mortality, length of stay (LOS), and cost of care associated with open surgical, laparoscopic, and radiology-guided percutaneous drainage in adult patients with infected WOPN.

Methods

Using the Nationwide Inpatient Sample (NIS), patients aged 18 years and older discharged with the diagnosis of WOPN between January 1, 2016 and December 31, 2016 who underwent open, laparoscopic, or percutaneous drainage were included. Patients’ characteristics including age, gender, and body mass index were reported. The primary endpoints were the mortality rate as well as length and cost of stay in each group. The secondary endpoint was the rate of procedural complications in each arm. Endpoints were reported and compared with studies assessing similar outcomes. Statistical Analysis System (SAS) statistical software (SAS Institute Inc., Cary, NC, USA) was used to perform the analysis.

Results

A total of 229 patients with the diagnosis of acute pancreatitis with infected necrosis were identified. Of these 229 patients, 27, 15, and 20 underwent open, laparoscopic, and percutaneous drainage, respectively. A total of eight studies were used for comparison of outcome variables. Mortality rate was found to be similar among comparison studies. LOS and costs varied widely among studies. There were significantly fewer pancreatic fistula and significantly more multi-organ failure complications as a result of open necrosectomy in the NIS study sample.

Conclusion

Overall, in analyzing the outcomes of patients undergoing intervention for infected WOPN through the 2016 NIS database, it appears that the database is representative of the majority of outcomes seen in similar clinical trials.

## Introduction

Acute pancreatitis (AP) accounts for over 275,000 hospital admissions in the United States, totaling healthcare costs that surpass 2.5 billion US dollars each year [1]. It is estimated that 15%-20% of AP episodes will be complicated by necrotizing pancreatitis, and 1%-9% of AP will develop into walled-off pancreatic necrosis (WOPN) [2,3]. Necrotizing pancreatitis is an encompassing term that includes both early-stage acute necrotic collection (ANC) as well as later stage WOPN. The latter represents an encapsulated collection of necrotic pancreatic or peripancreatic tissue that develops usually more than four weeks after the onset of acute necrotizing pancreatitis. WOPN can be asymptomatic, also referred to as sterile, in up to 50% of cases. However, when symptomatic or infected, WOPN is associated with significant morbidity and mortality [4]. Infected WOPN is differentiated from sterile WOPN by signs of clinical deterioration such as systemic infection or persistent organ failure, which not only requires more aggressive intervention but also results in an increased burden on the patient and hospital system [5,6]. While nutritional and medical support are the primary means by which sterile WOPN is treated, infected WOPN relies heavily on drainage by endoscopic, percutaneous, or surgical intervention [5,7].

Open surgical necrosectomy has been the standard of care for infectious complications of necrotizing pancreatitis up until recent years when minimally invasive surgery (MIS) challenged the open approach aiming at less morbidity, mortality, and use of hospital resources [8]. While open surgical necrosectomy provides a larger visual field to the abdomen, allowing for easier access to necrotic tissue, it also potentiates the risk of postoperative complications and has been shown to have inferior outcomes [9,10]. Recently published literature suggests that a step-up approach, consisting of laparoscopic or percutaneous drainage prior to open necrosectomy, may reduce mortality and the rate of complications in patients requiring surgical intervention [5,9]. However, without necrosectomy, minimally invasive strategies such as percutaneous or endoscopic drainage will successfully treat only 35%-55% of patients with infected WOPN [5]. The aim of this study was to describe the mortality, morbidity, length of stay (LOS), and cost of care for open surgical, laparoscopic, and radiology-guided percutaneous drainage in hospitalized adult patients with infected WOPN. An abstract for this study was submitted and accepted to DDW 2020, and aspects of this study have been published in the supplement section of *Gastroenterology*.

## Materials and methods

Study design and database description

This is a retrospective cohort study of adult patients hospitalized with WOPN who required surgical and/or radiological interventions at hospitals located across the United States in 2016. The Nationwide Inpatient Sample (NIS), the largest publicly available all-payer inpatient care database including information on nearly eight million inpatient hospital stays per year from roughly 1000 hospitals nationwide, was used to harvest data. The NIS provides estimates, both regional and national, of inpatient utilization, access, charges, quality, and outcomes. It represents a 20% stratified sample of all hospital discharges in the Unites States and is maintained by the Agency for Healthcare Research and Quality (AHRQ). The International Classification of Diseases, 10th revision, and Clinical Modification (ICD-10-CM) was used to identify patients who satisfy the inclusion criteria. The most recent version of the NIS database was released for data year 2017 and reflects diagnoses and procedure codes reported using ICD-10-CM. The present study qualified for exemption from institutional review board approval. 

Identification of patients

Using the NIS, patients aged 18 years and older who were discharged with the diagnosis of WOPN between January 1, 2016 and December 31, 2016 and who underwent pancreatic drainage - percutaneous and/or surgical (open or laparoscopic) - were included. Patients’ characteristics included age, gender, and body mass index. The following ICD-10-CM codes were used to identify patients with WOPN: K850.2, K851.2, K852.2, K853.2, K858.2, and K859.2 corresponding to idiopathic AP, biliary AP, alcohol-induced AP, drug-induced AP, other AP, and AP with infected necrosis, respectively. Patients were divided into those who underwent open necrosectomy (ICD-10-CM codes: 0F9G00Z, 0F9G0ZZ, 0FBG0ZZ), percutaneous drainage (ICD-10-CM codes: 0F9G30Z, 0F9G3ZZ, 0FBG3ZZ), and laparoscopic drainage (ICD-10-CM codes: 0F9G40Z, 0F9G4ZZ, 0FBG4ZZ).

Study outcomes and variables definition

The primary outcomes were the mortality rate, LOS, and cost of stay (COS) in each group. The secondary endpoint was the rate of procedural complications in each arm including bleeding or hematoma, blood product transfusion, acute respiratory failure, other respiratory complication, acute kidney injury, acute hepatic failure, perforation, and pancreatic fistula. Procedural complication rates were determined through the use of their respective ICD-10-CM codes listed in the NIS database appendix. The ICD-10-CM codes related to the complications have been listed in the Appendix. Multi-organ failure was defined by the presence of two or more ICD-10-CM codes attributed to the same patient. Mortality rates were determined by dividing the total number of patients who died during hospitalization by the total number in that respective procedural subgroup. COS was determined by total charges during patient’s hospitalization, not inclusive of professional fees. If sources provided total charges with professional fees, these fees were removed from the total charge amount. Length of stay was calculated by subtracting the admission date from the discharge date, with same day discharges corresponding to a LOS value of zero.

Comparison studies

A comprehensive literature search querying the PubMed database was conducted for manuscripts published between January 2010 and January 2020 that compared clinical outcomes of the procedures of interest as they related to infected WOPN. Only studies providing a contingency of data allowing for extrapolation of outcomes related to WOPN were included. Keywords in our search included open necrosectomy, MIS, infected WOPN, mortality rate, COS, LOS, and complications. The connector word “AND” was used to capture articles that were pertinent to our study. Articles eligible for inclusion were either retrospective (case-control studies) or prospective studies (randomized controlled trials) in the English language, conducted on human subjects. Comparison studies categorized as minimally invasive surgery/step-up approach (MIS/SUA) followed a primarily percutaneous drainage approach (“surgical step-up approach”) first, and these outcomes were compared with that of the percutaneous drainage arm of the NIS. Comparison studies analyzing COS were carried out in a country for which there is a publicly funded health system, making comparison analysis ineffective.

Statistical analysis

Categorical variables such as demographic information, mortality, and procedural complications were analyzed as percentages. Means and standard deviations were calculated to compare numeric variables. Statistical analysis was performed using χ^2 ^test to compare mortality and adverse events rates. P values were two-sided, and values less than 0.05 were considered statistically significant. Statistical analysis was performed using Statistical Analysis System (SAS) statistical software package version 9.4 (SAS Institute Inc., Cary, NC, USA).

Missing data and bias

Data that were substantially missing from the initial search, including that of comparison studies, were not included in the statistical analysis. Given the observational nature of this study, the STROBE (Strengthening the Reporting of Observational Studies in Epidemiology) checklist was used to analyze for bias and to assess study quality.

## Results

Patient characteristics

A total of 229 patients, identified with the primary diagnosis of AP with infected necrosis, were included (Table 1). Of these patients, 27 (11.7%) underwent open surgical necrosectomy, 15 (6.6%) laparoscopic drainage, and 20 (8.7%) percutaneous drainage. Males represented the majority in each study group, and the mean age among open surgical necrosectomy (55 ± 14 years), laparoscopic drainage (59 ± 12 years), and percutaneous drainage (57 ± 15 years) were reported. BMI data were collected, and the patients were subcategorized as having BMI greater than or equal to 30 or less than 30.

**Table 1:** Demographic Information of Treatment Groups Included From NIS Data 2016 WE: Weighted estimate; SD: standard deviation; BMI: body mass index.

Parameter	Open	Laparoscopic	Percutaneous
Number of patients (WE)	27 (135)	15 (75)	20 (100)
Number of female patients (WE)	9 (45)	6 (30)	6 (30)
Mean age ± SD	55 ± 14	59 ± 12	57 ± 15
BMI < 30 kg/m^2^	14.8%	20%	25%
BMI ≥ 30 kg/m^2^	25.9%	20%	15%

Inpatient mortality

The NIS 2016 study sample mortality ranged from 5% to 11% with open necrosectomy responsible for the highest in-hospital mortality (Table 2). For those undergoing open necrosectomy, mortality rate ranged from 10% to 33.3% with only the study by Woo et al. suggesting a lower mortality rate, although there was no significant difference found when comparing this data to that of the NIS 2016 data (p = 0.923) [11]. Mortality rate from the Rasch et al. study was 33.3%, which was significantly higher than that of the NIS sample (p = 0.046) [12]. For those undergoing MIS/SUA, mortality rate ranged from 0% to 18.8%. When comparing mortality data from the included studies to that of the NIS percutaneous treatment sample, no significant difference was seen.

**Table 2:** Comparison of Mortality Rate Among Studies Included in the Analysis ^+^As compared to NIS 2016 open necrosectomy data *As compared to NIS 2016 percutaneous drainage data MIS/SUA: Minimally invasive surgery/step-up approach; lap: laparoscopic; perc: percutaneous.

Mortality Data Among Comparison Studies		
Study	Mortality n (%)	p value^+^
Open necrosectomy		
Results of 2016 NIS sample study	3 (11.1%)	
van Santvoort et al. 2010	7 (15.5%)	0.598
Tan et al. 2014	3 (14.3%)	0.741
Woo et al. 2015	1 (10.0%)	0.923
Rasch et al. 2016	10 (33.3%)	0.046
Gomatos et al. 2016	28 (23.3%)	0.160
Wroński et al. 2017	6 (27.3%)	0.146
Study	Mortality n (%)	p value*
MIS/SUA		
Results of 2016 NIS data (lap, perc)	1 (6.0%), 1 (5.0%)	
van Santvoort et al. 2010	8 (18.6%)	0.151
Woo et al. 2015 (perc)	0 (0%)	0.172
Rasch et al. 2016	20 (10.5%)	0.205
Gomatos et al. 2016	42 (15.3%)	0.579
Wroński et al. 2017	9 (18.8%)	0.905
van Brunschot et al. 2018	6 (12.8%)	0.447

Length of stay and cost of stay

**Figure 1:**
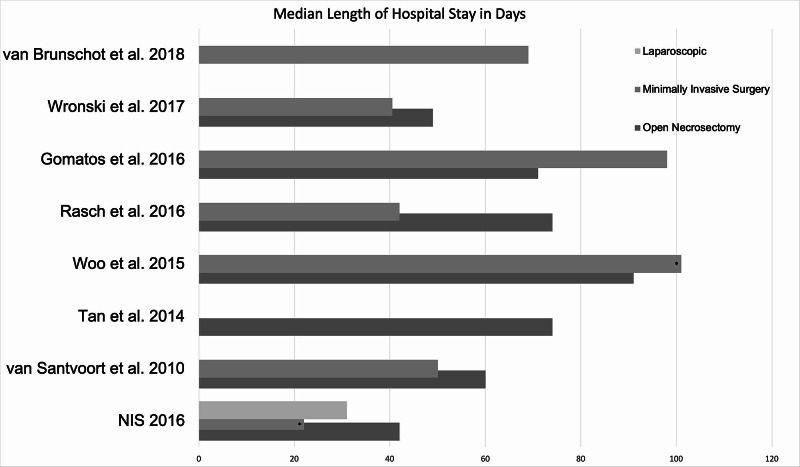
Comparison of Hospital Length of Stay Among Studies * indicates these parameters belong to the percutaneous drainage treatment arm of the respective studies; NIS 2016: results from our analysis of the NIS study sample. NIS: Nationwide Inpatient Sample.

**Figure 2:**
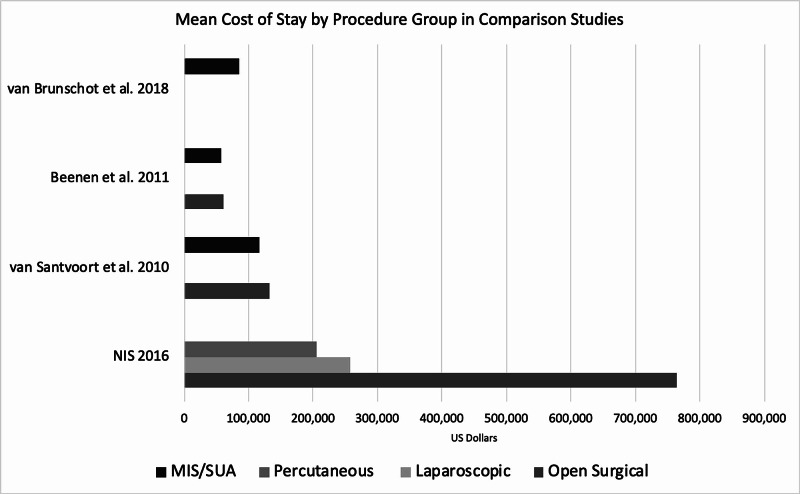
Comparison of Median Cost of Stay Among Included Studies MIS/SUA: Minimally invasive surgery/step-up approach; NIS 2016: results from our analysis of the NIS study sample; NIS: Nationwide Inpatient Sample.

Data pulled from the NIS 2016 showed that patients undergoing radiology-guided percutaneous drainage for WOPN were found to have a median length of hospital stay of 22 days, those undergoing laparoscopic drainage had a median LOS of 31 days, and those undergoing open necrosectomy had a median LOS of 42 days (Table 3). This data was then charted alongside comparison studies as seen in Figure 1. LOS for those undergoing open necrosectomy ranged from 42 to 91 days and those undergoing MIS/SUA ranged from 22 to 101 days, with the NIS sample accounting for the lowest LOS in both cases. From our analysis of 2016 NIS data, patients undergoing open surgical necrosectomy incurred the highest COS with an average of $764,428, while the COS for those undergoing laparoscopic drainage was $257,687 (Table 4). Patients undergoing percutaneous drainage spent the least amount on average, which amounted to $205,543 USD. Mean COS ranged from $60,913 to $764,428 for those undergoing open necrosectomy and $56,674 to $205,543 for those undergoing MIS/SUA. Comparison studies appear to show COS to be lower than that of the 2016 NIS data (Figure 2).

**Table 3:** Comparing Median Length of Hospital Stay of WOPN Studies (L): Laparoscopic procedures; (P): percutaneous procedures; WOPN: walled-off pancreatic necrosis.

Median Length of Hospital Stay in Days		
Study	Open Necrosectomy	Minimally Invasive Surgery
Results of 2016 NIS sample study	42	31 (L); 22 (P)
van Santvoort et al. 2010	60	50
Tan et al. 2014	74	-
Woo et al. 2015	91	101 (P)
Rasch et al. 2016	74	42
Gomatos et al. 2016	71	98
Wroński et al. 2017	49	40.5
van Brunschot et al. 2018	-	69

**Table 4:** Comparison of Median Cost of Stay Among Included Studies L: Laparoscopic; P: percutaneous; MIS/SUA: minimally invasive surgery/step-up approach.

Mean Cost of Hospital Stay (USD)		
Study	Open Surgical	MIS/SUA
Results of 2016 NIS sample study	764,428	257,687 (L); 205,543 (P)
van Santvoort et al. 2010	131,979	116,016
Beenen et al. 2011	60,913	56,674
van Brunschot et al. 2018	-	85,682

Complications and adverse events

Results of our analysis from the NIS 2016 sample suggest that those undergoing intervention for infected WOPN are likely to experience multi-organ failure with 52% of patient in the open necrosectomy arm, 53% in the laparoscopic arm, and 30% in the percutaneous arm experiencing this complication (Table 5). Complication rate for bleeding/blood transfusion ranged from 10% to 37% among those undergoing open necrosectomy, with the patients from the NIS 2016 data experiencing a significantly increased rate (37%) compared to those in the Gomatos et al. study (15%, p = 0.01) [13]. In the studies by Tan et al. and Gomatos et al., patients were less likely to experience multi-organ failure (17% and 25%, respectively, p = 0.04, p = 0.01) [14]. Additionally, patients receiving MIS/SUA from the Gomatos et al. study were less likely to be complicated by multi-organ failure compared to those undergoing percutaneous drainage in our analysis of the NIS data (12% and 30%, respectively, p = 0.02)[13]. Perforation was infrequent and was found to occur less often in those undergoing percutaneous drainage from the NIS data (0%) when compared to those undergoing the surgical step-up approach in the study by vanBrunschot et al. (17%, p = 0.049) [15]. Additionally, perforation occurred more frequently in those undergoing open necrosectomy in the NIS data than those in the Tan et al. study (18% and 0%, respectively, p = 0.04) [14]. Finally, pancreatic fistula occurred infrequently in those undergoing open necrosectomy from the NIS study sample (3%), which was found to be significantly less frequent than all studies in our analysis as detailed in Table 5. Of note, there was significantly fewer pancreatic fistulas seen in Gomatos et al. (5%) for those undergoing MIS/SUA as compared to those undergoing percutaneous drainage from the analysis done on the NIS 2016 data (20%, p = 0.01) [13].

**Table 5:** Comparison of Major Complications of Interventions by Study ^a^Statistically significant as compared to NIS 2016 open necrosectomy data ^b^Statistically significant as compared to NIS 2016 percutaneous drainage data ON: Open necrosectomy; L: laparoscopic; MIS/SUA: minimally invasive surgery/step-up approach. Complication data was not available for Rasch et al.

Major Complications of Interventions by Study					
Study	Procedure	Bleeding/Transfusion	Multi-organ Failure	Perforation	Pancreatic Fistula
Results of 2016 NIS data	ON	10 (37%)	14 (52%)	5 (18%)	1 (3%)
	L	5 (33%)	8 (53%)	1 (7%)	0 (0%)
	P	4 (20%)	6 (30%)	0 (0%)	4 (20%)
van Santvoort et al. 2010	ON	10 (22%) p = 0.17	18 (40%) p = 0.33	10 (22%) p = 0.71	17 (38%) p < 0.01^a^
	MIS/SUA	7 (16%) p = 0.72	5 (12%) p = 0.07	6 (14%) p = 0.08	12 (28%) p = 0.50
Tan et al. 2014	ON	3 (14%) p = 0.08	4 (17%) p = 0.02^a^	0 (0%) p = 0.04^a^	8 (38%) p < 0.01^a^
Woo et al. 2015	ON	1 (10%) p = 0.11	-	1 (10%) p = 0.53	4 (40%) p < 0.01^a^
	P	0 (0%) p = 0.17	-	1 (13%) p = 0.11	0 (0%) p = 0.17
Gomatos et al. 2016	ON	18 (15%) p = 0.01^a^	30 (25%) p = 0.01^a^	-	33 (12%) p = 0.01^a^
	MIS/SUA	50 (18%) p = 0.85	33 (12%) p = 0.02^b^	-	14 (5%) p = 0.01^b^
Wronski et al. 2017	ON	6 (29%) p = 0.47	12 (55%) p = 0.85	-	7 (32%) p = 0.01^a^
	MIS/SUA	8 (17%) p = 0.74	12 (25%) p = 0.67	-	10 (21%) p = 0.94
van Brunschot et al. 2018	MIS/SUA	10 (21%) p = 0.90	6 (13%) p = 0.09	8 (17%) p = 0.049^b^	13 (32%) p = 0.51

## Discussion

This is a unique study in that it compares morbidity and mortality as well as procedural complications in patients specifically with infected or symptomatic WOPN. The results confirm that WOPN is associated with a significant morbidity and mortality rate among patients requiring surgical intervention. While only half of the patients with WOPN will require surgical intervention, the findings of this study demonstrated the impact of the choice of intervention in managing infected WOPN on LOS and COS to the patient and healthcare system.

Open surgical necrosectomy was long considered the standard of care for patients with infected necrotizing pancreatitis. In 2010, the PANTER trial was the first randomized controlled trial to show improved morbidity and mortality in the use of a “step-up approach” with minimally invasive therapies compared to open necrosectomy and post-operative lavage in patients with infected or symptomatic pancreatic necrosis [9]. Since then, the step-up approach has become the standard of care. However, prior to the release of the Atlanta Classification in 2012, the term “walled-off pancreatic necrosis” had not been well-described, and few studies have since compared the outcomes of interventions for WOPN specifically [4]. Based on the results from the PANTER trial and the studies that followed, the 2013 International Association of Pancreatology and American Pancreatic Association (IAP/APA) guidelines for managing necrotizing pancreatitis suggest delaying intervention until the necrosis has become walled off when possible [10]. Relatively few studies have since compared morbidity and mortality data for patients with infected WOPN. The results of this study demonstrated a mortality rate of 11% from the NIS study sample and comparison rates between 10% and 33% for patients undergoing open necrosectomy for infected WOPN. This finding was consistent with previous studies comparing mortality outcomes in patients with infectious necrotizing pancreatitis with patients experiencing mortality rates of 5%-26% following open necrosectomy [10]. Similarly, a recent study by Husu et al. showed that patients undergoing surgical necrosectomy for WOPN have a 90-day mortality rate of 10.6%, which is comparable to the mortality rate seen in this study [16]. While no study in review of the literature has been able to find a statistically significant mortality benefit when comparing the different forms of intervention for infected WOPN, these results echo prior studies in that open necrosectomy is associated with a mortality rate of around 10% and that there may be evidence for improved survival with the use of MIS.

In comparing LOS in patients undergoing open necrosectomy, results of this study demonstrate that median LOS for the NIS sample is lower than that of comparison studies. These results were replicated when comparing LOS among the percutaneous drainage arm with those undergoing MIS/SUA from comparison studies. Additionally, patients with WOPN who underwent intervention in the United States during the year 2016 incurred an average bill of between $205,543 and $764,428 USD. In comparison studies, average cost of hospitalization appears to be much lower, although a direct comparison of these numbers is challenging, given that hospitalization charges are largely healthcare system-dependent and comparison studies were carried out in countries that have a publicly funded health system. Given the inability to extrapolate patient- and study-specific data from comparison studies, a complete statistical analysis was unable to be executed for COS and LOS. Interestingly, prior studies comparing COS and LOS for patients undergoing intervention for infectious necrotizing pancreatitis were unable to find a significant difference between open surgical necrosectomy and the more minimally invasive therapies. Primary contributors to COS include LOS (in particular, days in the intensive care unit), surgical personnel and costs, and complications that lead to extended hospital stay [17,18]. In a comparison study by Wronski et al., researchers suggest that ICU stay was significantly longer in patients undergoing open surgical necrosectomy compared to minimally invasive management, which may have contributed to the significantly increased cost and LOS observed in this study [19]. However, no ICU-specific data could be extracted from the NIS study sample. Another major contribution to cost and LOS in the all treatment arms was likely the rate of complications experienced by these patients. Open necrosectomy has been previously described as having a morbidity rate of up to 95% with complications of hemorrhage, wound infections, perforation, fistula formation, and organ failure [20]. Our analysis suggests that open necrosectomy in the NIS sample had a significantly lower rate of pancreatic fistula and a higher rate of multi-organ failure than comparison studies. This can likely be explained by the relatively small sample size included in the NIS data pull or the advancement in practice of performing the procedure. 

A systematic review of patients undergoing radiology-guided percutaneous drainage for infected pancreatic necrosis as an intervention alone showed successful treatment in nearly 56% of cases with a mortality rate of around 15.4% [21]. In light of this improved morbidity and mortality data, percutaneous drainage or endoscopic drainage is recommended as the first step in the step-up approach for the treatment of infectious pancreatic necrosis [22]. A review of the literature suggests that percutaneous drainage is complicated by internal or external pancreatic fistula formation in up to 20% of cases [20]. This study showed a similar 20% complication rate of pancreatic fistula formation in patients receiving percutaneous drainage for infected WOPN. Only the study by Gomatos et al. suggests a lower rate of pancreatic fistula formation for those undergoing MIS/SUA [15]. Additionally, this study showed a mortality rate of 5% in this study group and was similar to comparison studies. While the mortality rate range appears to be lower in those undergoing a surgical step-up approach, further prospective studies are required to better characterize any improvement in mortality seen for patients receiving percutaneous drainage for infected WOPN.

Given the administrative nature of the NIS database, the correct use of ICD-10 codes cannot be confirmed among the many hospitals sampled across the United States and constitutes a limitation of this study. Additionally, comparison studies that were retrospective in nature were unable to control procedural or outcome assessment, relying heavily on accurate clinical recordkeeping. These retrospective studies may involve cases that pre-date the Atlanta Classification for WOPN and IAP/APA guidelines for management of pancreatic necrosis, which further limits the ability to accurately compare all outcomes across studies [4,10]. Furthermore, the 2016 release of the NIS database, which was used in this study, does not include procedural coding for direct endoscopic necrosectomy (DEN), a treatment option that has recently shown superiority in the treatment of infected WOPN on its own. Most notably, patients undergoing DEN have been shown to have significantly fewer procedures to achieve clinical resolution, a decreased chance for procedural complications, and a significantly shorter LOS and decreased COS when compared to those undergoing the step-up approach for WOPN [23]. However, the NIS database is representative of practices across the entire United States, which makes the results of this study generalizable to the entire study population of interest.

## Conclusions

In conclusion, this study reiterates that infected WOPN is associated with significant morbidity, mortality, and utilization of patient and hospital resources. Mortality data from the 2016 NIS database suggests similar outcomes when compared with related studies. Additionally, mortality data among the minimally invasive/surgical step-up group was not found to be significantly different from those undergoing percutaneous drainage from the NIS database. While LOS appears to be shorter from the NIS data pull, there was a lack of available data to be extrapolated from comparison studies for analysis. Costs varied widely in this study, and comparable studies used data from publicly funded healthcare systems making it difficult to assess. There were however noticeable differences in some complications that may be attributed to the smaller sample size of the NIS or the advancement in clinical practice.

## Appendices

**Table 6:** Described Complications With ICD-10-CM Codes

Complication	ICD-10-CM Code
Bleeding/hematoma	D62, R58, K92.2, K92.1, K91.61, K91.62, K91.870, K91.871, D78.02, D78.22, K91.61, K91.62, K91.840, K91.841, L76.22, M96.811, M96.831
Blood product transfusion	30243N0 30243N1 30243P0 30243P1 30243H0 30243H1 30240N0 30240N1 30240P0 30240P1 30240H0 30240H1 30230H0 30230H1 30230N0 30230N1 30230P0 30230P1 30233N0 30233N1 30233P0 30233P1
Acute respiratory failure	J96.01, J96.00, J95.821, J96
Other respiratory complication	J9562 J9561 J9572 J9571 J9588 J95861 J95860 J95831 J95830 J95863 J95862 J9589 J95821 J95822
Acute kidney injury	N17.9, N17.8, N99.0, N17.0
Acute hepatic failure	K72.01, K72.00, K91.82, K72.0
Perforation	K22.3, K63.1, K82.2, K83.2, T 85.59
Pancreatic fistula	K86.8, K86.9
